# 

**Published:** 1892-02

**Authors:** 


### THE AMERICAN JOURNAL OF DENTAL SCIENCE.

PUBLISHED MONTHLY.

$2.50 PER ANNUM.

EDITED BY

F. J. S. GORGAS, M. D., D. D. S.
RICHARD GRADY, M. D., D. D. S.

Vol. 25. THIRD SERIES No. 10.

BALTIMORE:

SNOWDEN & COWMAN, 9 W. Fayette Street.

LONDON:
TRUBNER & CO., 60 Paternoster Row

Entered at the Post Office at Baltimore, Md., as Second-class Matter.

PAYABLE IN ADVANCE.

